# Simultaneous Quantification of Four Ginsenosides in Rat Plasma and Its Application to a Comparative Pharmacokinetic Study in Normal and Depression Rats Using UHPLC-MS/MS

**DOI:** 10.1155/2021/4488822

**Published:** 2021-08-23

**Authors:** Lian-yun Du, Tao Jiang, Kun Wei, Shuang Zhu, Yan-long Shen, Ping Ye, Hui-e Zhang, Chang-bao Chen, En-peng Wang

**Affiliations:** ^1^Jilin Ginseng Academy, Changchun University of Chinese Medicine, Changchun 130117, Jilin, China; ^2^Affiliated Hospital of Changchun University of Chinese Medicine, Changchun University of Chinese Medicine, Changchun 130117, Jilin, China

## Abstract

A sensitive method has been developed for simultaneous determination of ginsenoside Rh_1_ (G-Rh_1_), ginsenoside Rb_1_ (G-Rb_1_), ginsenoside Rc (G-Rc), and ginsenoside Rd (G-Rd) in rat plasma of normal and depression model group after oral administration of their solutions by using Ultra-High-Performance Liquid Chromatography-Tandem Mass Spectrometry (UHPLC-QQQ-MS). The biological samples were prepared by protein precipitation. Ginsenoside Rg_3_ (G-Rg_3_) was used as an internal standard (IS). MS analysis was performed under the multiple reaction monitoring (MRM) with electron spray ionization (ESI) operated in the negative mode. The method showed good linearity over a wide concentration range (*R*^2^ > 0.999) and obtained lower limits of quantification (LLOQ) of 5 ng/mL. The whole analysis procedure could be completed in as short as 16.5 min. The intraday precisions, interday precisions, and stabilities were less than 10%. The extraction recoveries from rat plasma were exceeded 86.0%. The results indicated that there were significant differences between the two groups on pharmacokinetics parameters; the absorptions of four analytes in the depression group were higher than those in the normal group because the liver metabolism and internal environment of the model rats had been affected.

## 1. Introduction

Depression is a sort of chronic mental disorder characterized by persistent black mood, which seriously affects the physical and mental health of human race [1]. Up to now, a large number of studies illustrated that a certain relationship was observed between the incidence of depression and the maladjustment of Hypothalamus-Pituitary-Adrenal (HPA), and excessive stress stimulation was considered to be the major cause of depression occurrence [2–5]. A natural stressful stimulus such as UVB radiation can trigger the activation of the HPA axis [6], no matter what acute or chronic dose irradiation may both individually reduce neurogenesis and synaptic protein expression, which finally lead to a depression-like behavior [7, 8].

At present, conventional chemical medicines on depression usually have various limitations or side effects, such as sleep disorders, cognitive dysfunction, high recurrence rate, and even much more severe effects [9–11]. Sometimes, as a complementary and alternative way, we often turn our eyes toward the traditional medicine (TM). TM has the advantages of much fewer side effects and lower toxicity, as well as higher efficacy and plenty of cured cases in the area of depression [12]. Ginseng, one of the most popular plants in East Asia, Europe, and North America, enjoys its reputation of “The king of herbs” [13–15]. Ginsenosides, widely considered as the main active ingredients, show a variety of pharmacological activities including increasing the ability to resist pressure, stimulating immune function, and protecting the nervous system and were responsible for the anti-inflammation function, antioxidation, and immunomodulatory effect [16, 17]. Lately, some up-to-date studies have demonstrated that ginseng has a huge potential in antidepression treatment procedures, and many active ingredients from the herb have a chance to be the candidate drugs [18–20]. It has been proved that some PPD-type ginsenosides including G-Rb_1_, G-Rd, and partial PPT-type ginsenosides have antidepressant and neuroregulatory effects and other PPD-type ginsenoside such as G-Rc may participate in the regulation of neuroprotection [21–25].

UHPLC-MS/MS has been widely focused on the preclinical analytical application research and pharmacokinetics during the last decade [26]. Small particle sizes in columns in UHPLC systems play a crucial role in the process of separation, which can not only increase separation efficiency and sensitivities but also reduce the chromatographic run time [27]. Thus, the purpose of this article was to set up a sensitive, selective, and accurate method to simultaneously performing the quantitation analysis on G-Rh_1_, G-Rb_1_, G-Rc, and G-Rd in rat plasma. It was expected to reveal how depression affects the absorption and metabolism on those active substances mentioned above through the pharmacokinetic parameter comparison between normal and depressed rats. It was expected that the results of this study will provide a broader idea for future antidepression studies on ginseng and the simultaneous determination methodology via mass spectrometry on ginsenosides.

## 2. Materials and Methods

### 2.1. Chemicals, Reagents, and Animals

G-Rh_1_, G-Rb_1_, G-Rc, G-Rd, and G-Rg_3_ (purity >99%) were purchased from the National Institute for the Control of Pharmaceutical and Biological Products (Beijing, China). HPLC-grade methanol and acetonitrile were purchased from Fisher Scientific (Waltham, Massachusetts, USA). All other chemicals in the experiment were analytical grade. The water (18.2 M) used in the experiment was doubly distilled water, and it was obtained from a laboratory water purification system (HHitech, China).

48 male Wistar rats (6∼8 weeks old, 180 ± 20 g) were purchased from Liaoning Changsheng Biotechnology Co., Ltd. (Benxi, China), and their certification number was 2631260011107325. Rats were housed in a squirrel cage in a house with a temperature of 22 ± 2°C and relative humidity of 50% ± 2%. All animals received food and water ad libitum. The animals were acclimatized to the house for 3 days prior to the experiment. All the experimental procedures were performed according to the Guide for the Care and Use of Laboratory Animals and related ethics regulations of Changchun University of Chinese Medicine.

After 3 days of acclimatization in facilities, 48 rats were randomly divided into two groups with 24 rats in each group: one group was the normal group, and the other group was the depression model group. The normal group did not undergo any treatment during the modeling process, the depression model group was induced by ultraviolet radiation B (UVB). The shaved back of the Wister model rat was 30∼42 cm from the light source and exposed to 366 mJ·cm^−2^ of UVB for 5 days in a homemade box. After the 6th day, the animals were irradiated once for 2 days for a total of 22 days (14 times). The total radiation dose was 5.12 J·cm^−2^. After the successful establishment of the model, the normal group and the depression model group were randomly divided into four groups, respectively. Four groups of rats in the normal group were taken orally with different analytes, respectively, including oral administration of G-Rh_1_, G-Rb_1_, G-Rc, and G-Rd, same for the depression model group.

### 2.2. Instruments and UHPLC-MS/MS Conditions

The UHPLC-MS/MS system consisted of a Thermo Ultimate 3000 system and TSQ ENDURA mass spectrometer using an electrospray ionization (ESI) and X Calibur data processing system (Thermo, USA). Optimized MS parameters were as follows: drying gas temperature, 350°C; ion transport tube temperature, 325°C; gasification temperature, 275°C; atomizing gas pressure, 255 kPa; sheath gas, 35 psi; aux gas, 10 psi; sweep gas, 2 psi; collision voltage, 3500 V; the cracking voltage, 175 V; and the tapered voltage, 65 V. The injection volume was 5 *μ*L, and the total run time was 16.5 min. Some parameters of MRM are shown in Table 1.

The mass spectrometer was operated by the negative multiple reaction monitoring (MRM) mode. Chromatography was achieved on an Ascentis® Express C_18_ column (5.0 mm × 3.0 mm, 2.7 *μ*m) from Sigma-Aldrich (St. Louis, MO, USA). The gradient elution mobile phase was composed of 0.1% formic acid-purified water in phase A and acetonitrile in phase B. The gradient program was as follows: 0∼2 min, 20%B; 2∼5 min, 20%B⟶35%B; 5∼10 min, 35%B⟶41%B; 10∼13 min, 41%B⟶80%B; 13∼14 min, 80%B⟶20%B; and 14∼16.5 min, 20%B. The flow rate was set at 0.3 mL/min, and the temperature was kept at 35°C.

### 2.3. Behavior Testing

#### 2.3.1. Sucrose Preference Test

In order to see if the rats were prone to depression, the sucrose preference test was applied. We referred to the method in [28] and improved it appropriately. Before starting the test, rats were kept in quiet rooms and adapted to the sucrose solution. First, we prepared two bottles of 1% sucrose solution for rats to drink for 24 h, followed by one bottle of 1% sucrose solution and one bottle of pure water for 24 h, and changed the positions of the two bottles at the intermediate point of time. Rats were banned drinking water for 24 h, and their preference was tested for sucrose solution. In this experiment, all rats were kept in a single cage for 24 h and prepared with two bottles of water which were weighed before the experiment, and the amount of sucrose solution the rats drank was calculated by the difference between weight before and after the test. The sucrose preference was calculated as follows: sucrose preference (%) = sucrose consumption (g)/(sucrose consumption (g) + pure water consumption (g)) × 100%.

#### 2.3.2. Forced Swimming Test

Each rat was placed into a cylindrical container; the container was 11 cm in diameter, 25 cm in height, and 20 cm in the depth of the water (25 ± 1 °C). The test duration was 6 min, and the time of the experiment was recorded by two trained students [29]. The rat was quiescent, and the body did not struggle as a standard of immobility. After the experiment, the rats were dried with a towel.

#### 2.3.3. Open-Field Test

This test is intended to test if the animals exhibit a depression-like behavior, using a square box (100 × 72 × 40 cm) and a mobile phone. Rats were put in the center of the box, respectively, followed by mobile phone shooting and timekeeping at the same time, which stopped after 5 min. The number of horizontal movements and the number of vertical movements were recorded by using the mobile phone, respectively [29]. After testing each rat, the inside and bottom of the box were thoroughly cleaned with alcohol so as not to affect the next test result.

### 2.4. Preparation of Stocks, Calibration Samples, and Quality Control Samples

The stock solutions of G-Rh_1_, G-Rb_1_, G-Rc, G-Rd, and IS were prepared separately in methanol at the final concentration of 1.0, 1.0, 1.0, 1.0 mg·mL^−1^, and 1.5 *μ*g/mL, respectively, and then, the standard working solutions were prepared by the stock solutions which were diluted with methanol. The IS working solution was also prepared by diluting the IS stock solution with methanol. The calibration curve standard samples were prepared by spiking 100 *µ*L of the working solution into 100 *µ*L of rat blank plasma at final concentrations of 5–10000 ng·mL^−1^ of G-Rh_1_, G-Rb_1_, G-Rc, and G-Rd. Then, each sample was spiked with 100 *µ*L of the IS solution and 100 *µ*L of the methanol solution, vortexed for 3 min, and centrifuged at 12000 r/min for 10 min. Finally, the supernatant was filtered through a 0.22 *µ*m microporous membrane and all the solutions were stored at 4°C before analysis. The blank control was prepared with 100 *μ*L rat blank plasma, and then, the abovementioned steps were completed. Three levels (25, 250, and 2500 ng/mL) of quality control (QC) samples were prepared by spiking standard working solutions into blank plasma.

### 2.5. Sample Preparation

All the plasma samples were stored at −20°C and thawed at room temperature immediately before the experiment. Each plasma sample (100 *μ*L) was added to a 1.5 mL tube and then proceeded as per the method under Section 2.4.

### 2.6. Method Validation

According to the Food and Drug Administration guidelines, we validated the established method.

#### 2.6.1. Specificity

In order to verify the specificity of the method, we compared the chromatograms of rat blank plasma, rat blank plasma spiked with four analytes and IS and rat plasma sample 1 h after administration of four analytes at a dose of 80 mg/kg.

#### 2.6.2. The Linearity of Calibration Curves

The linearity of the assay for rat plasma was assessed by analyzing the calibration curves using least-squares linear regression of the peak area ratios of the analyte to the IS versus the nominal concentration of the calibration standard with a weighed factor (1/*x*^2^). The minimum concentration of the standard curve was taken as the lower limit of quantification (LLOQ).

#### 2.6.3. Intraday Precision, Interday Precision, and Accuracy

In order to evaluate the intraday precision, interday precision, and accuracy, we analyzed three concentration levels (25, 250, and 2500 ng/mL) of the QC plasma samples with six replicates at the same day and 3 continuous days, respectively, with the relative standard deviation (RSD) for precision and relative error (RE) standards for accuracy.

#### 2.6.4. Recovery and Matrix Effect

In order to evaluate the recoveries of G-Rb_1_, G-Rc, G-Rd, and G-Rh_1_, we compared the peak area of QC (25, 250, and 2500 ng/mL) samples with the corresponding concentration spiked into the blank plasma sample. In order to evaluate the matrix effects, we compared the peak areas of QC samples with the areas of the corresponding concentration standard solutions. IS was determined in the same manner.

#### 2.6.5. Stability

For confirming the stability of four analytes, we analyzed QC samples with three replicates under the following conditions: stability of short term (samples stored at room temperature for 24 h); stability of long term (samples stored at −20°C for 15 days); and stability of freeze/thaw (3 cycles, −20°C, room temperature).

### 2.7. Method Application

#### 2.7.1. Pharmacokinetic Study

24 rats of the model group were induced into the depressive model by the previously described method. The animals were fasted for 12 h with free access to water prior to the experiment; then, the normal and depression model group of rats were assigned to receive G-Rh_1_, G-Rb_1_, G-Rc, and G-Rd by oral administration of their solutions (suspended in 0.5% CMC-NA), respectively. In addition, 80 mg/kg was selected as the oral administration dose according to the previous laboratory studies and the requirements of experiments [30]. Blood samples (about 250 *μ*L) were obtained via the rats' orbital vein at 0.08, 0.17, 0.33, 0.5, 1.0, 2.0, 4.0, 6.0, 8.0, 12, 24, 36, 48, and 72 h after oral administration and were collected into heparinized centrifuge tubes. The blood samples were cooled naturally for 30 minutes and centrifuged at 3000−r/min for 10 min. The plasma samples obtained were stored at −80°C until the experiment.

#### 2.7.2. Pharmacokinetic Data Analysis

The pharmacokinetic parameters of four components obtained in this experiment are mainly as follows: area under the time concentration curve (AUC_0−*t*_, AUC _0−∞_), the peak concentration (*C*_max_), mean residence time (MRT), elimination half-life (*T*_1/2_), the peak time (*T*_max_), clearance rate (CLz/F), and so on. Two-compartmental analysis was used to calculate the relationship between the plasma concentration versus time data of four ginsenosides by using the DAS 3.0 software package (Chinese Pharmacological Society). Statistical analysis between the two groups was dealt with SPSS 21 using a One-Way ANOVA analysis. Data were expressed as mean ± SD, and the *p* value less than 0.05 was considered to be significantly significant.

## 3. Results and Discussion

### 3.1. Rats Depression Model Was Successfully Established

**Figure 1:**
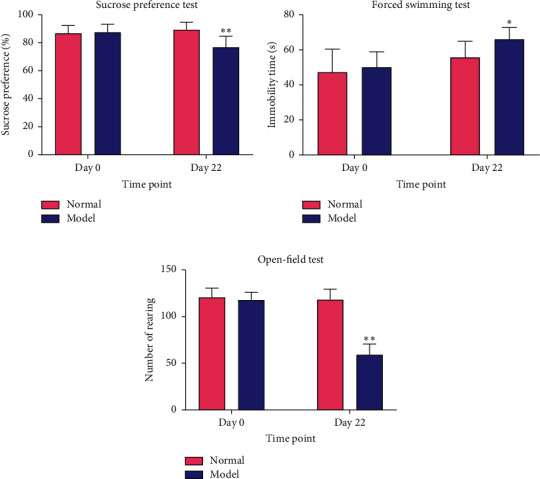
The successful construction of the rat depression model: the sucrose preference of rats in the sucrose preference test (a); the immobility time of rats in the forced swimming test (b); the number of rearing of rats in the Open-field test (c). The values were expressed as the mean ± SD for *n* = 6. ^*∗*^*p* < 0.05, ^*∗∗*^*p* < 0.01.

In order to confirm whether the rat depression model was successfully established or not, we verified a series of indicators, such as the sucrose preference test, forced swimming test, and the open-field test. For the sucrose water consumption, there was no significant difference between the normal and the depression model group (*p* > 0.05) on day 0 (before stimulation). By contrast, the sucrose water consumption of the normal group was significantly higher than the depression group on the 22nd day after treatments (*p* < 0.01). No significant difference was observed between the two groups during the forced swimming test, day 0 before the stimulation (*p* > 0.05), while the immobility time of the depression group was significantly longer than the normal group on the 22nd day after the beginning of the experiment (*p* < 0.05). It was shown no significant difference between groups on day 0 (*p* > 0.05); however, the number of rearing of the depression group was significantly lower than the normal group by the end of the 22-day treatment periods in the open-field test (*p* < 0.01) (Figure 1). In conclusion, the establishment of the depressed model was successful.

### 3.2. Optimization of UHPLC-MS/MS Conditions

**Figure 2:**
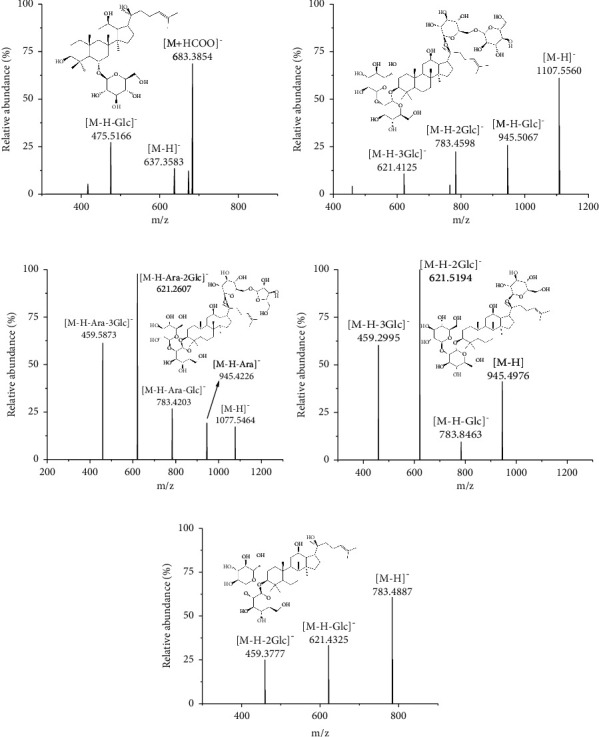
Chemical structures and product ions of G-Rh1 (a), G-Rb1 (b), G-Rc (c), G-Rd (d), and IS (e).

In order to obtain chromatographic analysis performance with better separation and retention time, two mobile phases, acetonitrile and methanol, were evaluated. The results showed that acetonitrile had better separation efficiency than methanol, which was more suitable for this experiment. 0.1% formic acid was added to the water as the mobile phase, and the chromatographic peak shape of IS and four analytes was more beautiful. Referring to the test, mass spectrometry analysis of IS and four analytes was performed using 0.1% formic acid-acetonitrile water as the mobile phase for the process of gradient elution. The results show that several ginsenosides are ionization in a negative ion mode, and the mass spectrum of five ginsenosides is shown in Figure 2.

### 3.3. Method Validation

#### 3.3.1. Specificity

**Figure 3:**
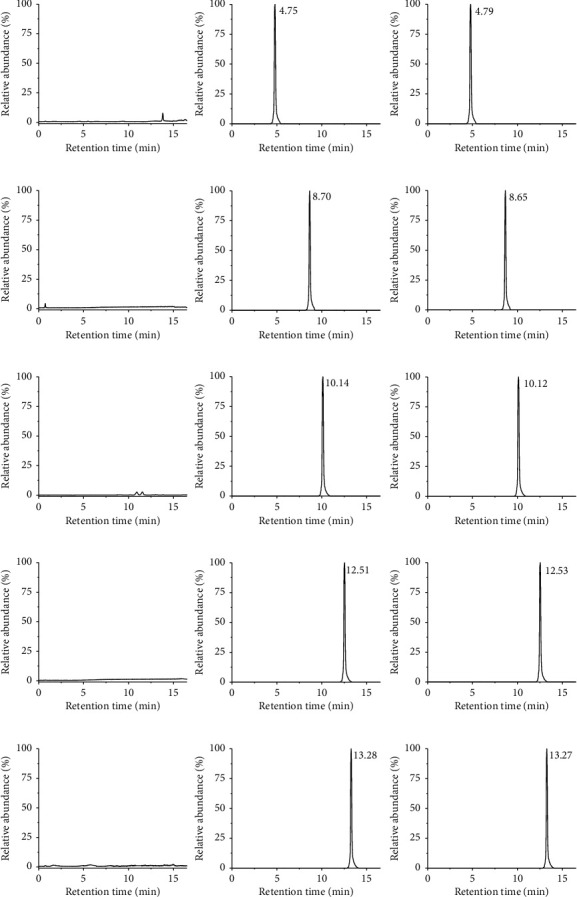
Representative multireaction monitoring mode chromatogram of G-Rh_1_ (a), G-Rb_1_ (b), G-Rc (c), G-Rd (d), and IS (e) in blank plasma (A), blank plasma spiked with four analytes and IS (B), and plasma sample 1 h after oral administration at a dose of 80 mg/kg (C).

As shown in Figure 3, it was observed that the retention time of endogenous did not interfere with the determination of the four analytes and the internal standard. The method showed good selectivity and baseline separation. The retention time was 4.75 min, 8.70 min, 10.14 min, 12.51 min, and 13.28 min, respectively.

#### 3.3.2. The Linearity of Calibration Curves

The linear relationship of the calibration curve was established by plotting the relationship between peak area ratios and the concentration of the four analytes. The four analytes exhibited good linearity with correlation coefficients (*R*^2^ > 0.999). The LLOQ of the four analytes was lower than 5 ng/mL, 5 ng/mL, 5 ng/mL, and 5 ng/mL, respectively, which met the quantitative requirements (Table 2).

#### 3.3.3. Intraday Precision, Interday Precision, and Accuracy

Table 3 indicated the precision and accuracy of the method. The results presented that the precisions and accuracies of all the four analytes are good and acceptable (RSD ≤9.69%, −8.92% ≤ RE ≤ 7.70%).

#### 3.3.4. Recovery and Matrix Effect

As shown in Table 3, the recoveries of four analytes at three concentrations were 86.06%∼96.86% and the matrix effects of them were 87.37%∼97.57. Meanwhile, the recoveries and the matrix effects of IS were 93.76 ± 3.28% and 95.12 ± 2.90%, indicating that the method was satisfactory.

#### 3.3.5. Stability

As shown in Table 4, the RE and RSD values of four analytes range from -9.34 to 8.15, and it can be seen from the abovementioned results that four analytes in plasma remained stable under different storage conditions, and the results met the requirements of this experiment.

### 3.4. Pharmacokinetic Application and Discussion

**Figure 4:**
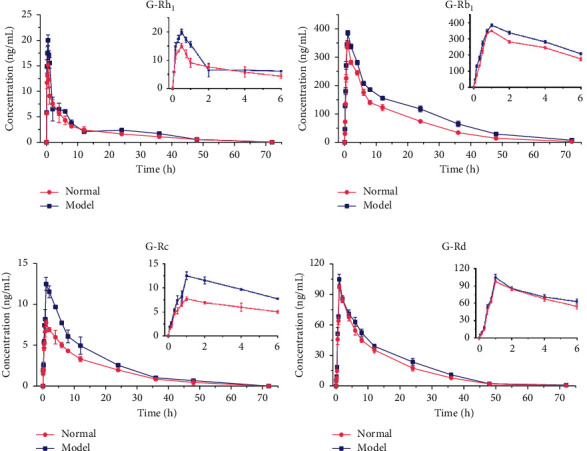
Mean plasma concentration-time profiles of compounds in rat plasma after oral administration of G-Rh_1_ (a), G-Rb_1_ (b), G-Rc (c), and G-Rd (d) at a dose of 80 mg/kg (mean ± SD, *n* = 6).

In this study, it was verified that the UHPLC-MS/MS method had been successfully applied to the quantitative analysis in rat plasma after oral administration of G-Rh_1_, G-Rb_1_, G-Rc, and G-Rd solutions to individual rats (*n* = 6) in the normal and depressed model. According to the F-test and Akaike's information criterion, a two-compartment PK model fitted the plasma data of the ginsenosides in the normal and depression model group of rats. The calculated PK parameters were *C*_max_, *T*_max_, AUC_0−*t*_, AUC _0−∞_, *T*_1/2_, CLz/F, and MRT, and they are summarized in Table 5. The mean plasma concentration-time curve is presented in Figure 4.

According to the pharmacokinetic data between the two groups of rats, as Table 5 shows, *C*_max_ of G-Rh_1_, G-Rb_1_, and G-Rc increased remarkably compared with the normal group (*p* < 0.05), while the G-Rd concentration increased but not significantly. AUC had a similar phenomenon, G-Rh_1_, G-Rb_1_, and G-Rd increased significantly, but G-Rc was not. In a word, we could easily find that the AUC values and *C*_max_ values in the depression model group were increased and Clz/F was decreased as compared with the normal group, and the bioavailability of ginsenosides in the depression model could be improved. Liver metabolism can be influenced by a variety of external factors, including typical psychosocial factors such as depression [31]. The depression induced by UVB radiation was able to change the internal environment of rats by producing oxidative damage to hepatocytes which subsequently affect their normal metabolism in the liver [32–34]. However, the activities of CYP450 enzymes in liver microsomal can be enhanced by UVB-induced depression in the previous report. As a result, it can be easily speculated that the increased blood concentration of four ginsenosides may have some connection with inhibiting the activities of CYP450 enzymes to show their potential efficacy of antidepressant effect [32]. Among the four analytes, G-Rb_1_ showed the largest value of AUC, and previous studies reported the potential efficiency of the antidepressant of G-Rb_1_ [35]. Also, it had been reported that both G-Rb_1_ and G-Rc can improve the activities of total glutathione (GSH) and superoxide dismutase (SOD) activities in cells after UVB irradiation [36, 37]. In the previous study, it had been found that oral administration of G-Rd could significantly alleviate depression by stimulating the NF-*κ*B-mediated BDNF expression in the hippocampus of rat [23]. G-Rh_1_ was rapidly absorbed in the rat plasma with an average Tmax of about 0.5 h, while others reached their maximum plasma concentrations at the time point of about 1 h. The differences between the two types could be ascribed to the widespread excretion of the PPD type in the renal and biliary tract more slowly than that of the PPT type [38].

## 4. Conclusions

To sum up, a new rapid UHPLC-MS/MS method was set for simultaneous determination of ginsenosides in rat plasma after oral administration in this study. The developed method was successfully applied to compare the pharmacokinetic behaviors between normal and depression model group of rats after oral administration of G-Rh_1_, G-Rb_1_, G-Rc, and G-Rd solutions. According to the results of this experiment, the absorption of the four ginsenosides in the normal group was not as good as that in the depression model group. It was speculated that the pathological status may affect the pharmacokinetic characteristics of rats in vivo. This method has been successfully applied to the pharmacokinetic study of ginsenosides with good specificity, precision, accuracy, recovery, and stability, which can provide new ideas and methods for the pharmacokinetic study of ginsenosides in vivo in the future.

## Figures and Tables

**Table 1:** Optimized parameters for the detection of G-Rh_1_, G-Rb_1_, G-Rc, G-Rd, and IS.

Analytes	Retention time (min)	Detect mode	Precursor	Product	Collision energy (eV)	RF lens (V)	Fragmentor voltage (V)
G-Rh_1_	4.75	ESI	637.3583	475.5166	40.287	198.371	175
G-Rb_1_	8.70	ESI	1107.5560	945.5067 783.4598 621.4125	55.000	259.652	220
G-Rc	10.14	ESI	1077.5464	945.4226 783.4203 621.2607 459.5873	49.792	247.820	175
G-Rd	12.51	ESI	945.4976	783.8463 621.5194 459.2995	45.646	276.337	220
G-Rg_3_ (IS)	13.28	ESI	783.4890	621.4325 459.3777	41.449	298.483	175

**Table 2:** The linearities for G-Rh_1_, G-Rb_1_, G-Rc, and G-Rd (*n* = 3).

Analytes	Calibration curves	Linear range (ng/mL)	*R*2	LLOD (ng/mL)	LLOQ (ng/mL)
G-Rh1	*y* = 0.0006*x* + 0.0015	5–10000	0.9997	1.5	5
G-Rb1	*y* = 0.0001*x* + 0.0005	5–10000	0.9993	1.5	5
G-Rc	*y* = 0.0002*x* + 0.0010	5–10000	0.9999	1.5	5
G-Rd	*y* = 0.0005*x* + 0.0028	5–10000	0.9999	1.5	5

**Table 3:** Summary of intraday and interday precisions, accuracies, extraction recoveries, and matrix effects of the four analytes in rat plasma (*n* = 6).

Analytes	Concentration (ng/mL)	Intraday RSD (%)	Interday RSD (%)	Accuracy (RE%)	Recovery (%)	Matrix effect (%)
G-Rh_1_	25	9.43	9.69	0.58	90.65 ± 2.34	95.67 ± 2.45
	250	0.95	0.49	3.84	93.67 ± 4.08	87.37 ± 8.45
	2500	1.21	2.95	4.08	96.59 ± 0.62	97.57 ± 3.11

G-Rb_1_	25	6.65	6.21	−3.47	86.06 ± 2.57	91.33 ± 0.96
	250	0.98	4.56	−1.44	91.86 ± 2.25	94.22 ± 1.47
	2500	3.50	2.38	2.38	94.19 ± 1.55	95.55 ± 1.22

G-Rc	25	5.35	6.21	−7.44	89.46 ± 5.13	89.42 ± 3.17
	250	4.38	3.28	6.34	91.28 ± 4.56	94.43 ± 5.85
	2500	3.86	4.61	−6.51	93.60 ± 2.02	96.85 ± 7.33

G-Rd	25	6.29	4.82	−8.92	87.64 ± 4.87	91.78 ± 5.38
	250	1.65	2.20	−5.12	90.87 ± 0.56	96.65 ± 3.97
	2500	3.87	4.51	7.70	96.86 ± 5.08	89.94 ± 3.77

**Table 4:** Stabilities of G-Rh_1_, G-Rb_1_, G-Rc, and G-Rd under various storage conditions (*n* = 6).

Analytes	Concentration (ng/mL)	Short term (24 h, room temperature)		Long term (15 days, −20°C)		Freeze-thaw (3 cycles)		Postpreparative (8 h, 4°C)	
		RE (%)	RSD (%)	RE (%)	RSD (%)	RE (%)	RSD (%)	RE (%)	RSD (%)
G-Rh_1_	25	4.78	3.69	−3.78	0.58	1.04	4.13	5.46	1.65
	250	−4.64	4.57	−7.34	7.66	3.48	3.62	3.87	3.64
	2500	3.75	2.56	6.66	5.93	4.86	0.78	5.19	4.79

G-Rb_1_	25	1.76	4.87	1.22	6.21	5.87	4.42	5.55	7.03
	250	−2.24	1.02	2.22	0.75	−9.34	2.77	2.74	3.25
	2500	2.08	3.62	−3.06	4.24	3.98	2.95	−7.34	1.58

G-Rc	25	−3.43	4.02	4.43	4.89	−6.43	4.93	−4.39	5.63
	250	−6.34	4.38	6.32	7.36	−5.65	4.7	4.53	1.07
	2500	2.08	1.66	8.15	3.18	4.01	4.89	3.75	0.73

G-Rd	25	3.98	5.42	4.83	3.28	5.43	3.34	−6.3	5.79
	250	−7.43	0.72	−1.37	3.33	−8.43	4.72	7.64	3.85
	2500	−3.87	2.09	2.65	3.23	−1.75	4.05	3.85	0.17

**Table 5:** Pharmacokinetic parameters of the four analytes in the normal and model group.

Parameters	G-Rh_1_	G-Rb_1_	G-Rc	G-Rd
*Normal*				
*C*_max_ (mg/L)	15.163 ± 0.815	349.840 ± 6.238	7.741 ± 0.366	97.458 ± 1.800
*T*_max_ (h)	0.50 ± 0.01	1.00 ± 0.01	1.00 ± 0.01	1.00 ± 0.01
AUC _0-72_ (mg/L^*∗*^h)	128.871 ± 68.509	10399.870 ± 5196.010	228.760 ± 110.731	2061.658 ± 1011.618
AUC _0-∞_ (mg/L^*∗*^h)	129.100 ± 68.507	10585.975 ± 5201.551	229.541 ± 110.102	2062.555 ± 1011.536
*t*_1/2_ (h)	10.731 ± 0.905	17.774 ± 0.207	9.659 ± 1.093	9.631 ± 0.206
CLz/F (L/h/kg)	621.227 ± 54.664	17.096 ± 0.382	647.808 ± 36.107	64.895 ± 2.255
MRT (h)	22.278 ± 10.621	15.952 ± 0.851	16.111 ± 2.798	13.997 ± 0.390

*Model*				
*C*_max_ (mg/L)	20.010 ± 1.027^*∗∗*^	385.694 ± 8.339^*∗*^	12.497 ± 0.809^*∗∗*^	104.959 ± 5.034
*T*_max_ (h)	0.50 ± 0.01	1.00 ± 0.02	1.00 ± 0.01	1.00 ± 0.03
AUC _0–72_ (mg/L^*∗*^h)	148.939 ± 62.329^*∗*^	26347.000 ± 13042.423^*∗*^	295.337 ± 165.908	2583.439 ± 1254.680^*∗*^
AUC _0-∞_ (mg/L^*∗*^h)	149.172 ± 62.330^*∗*^	29419.304 ± 12850.460^*∗*^	295.641 ± 166.081	2587.285 ± 1253.860^*∗*^
*t*_1/2_ (h)	9.765 ± 0.825	13.472 ± 0.588^*∗∗*^	8.166 ± 0.881	10.198 ± 0.511
CLz/F (L/h/kg)	544.957 ± 6.150	11.994 ± 0.385^*∗*^	450.788 ± 23.974	55.744 ± 2.366^*∗∗*^
MRT (h)	16.589 ± 1.745	19.683 ± 0.793^*∗*^	14.639 ± 0.190	15.126 ± 0.671

Data were reported as mean ± SD for *n* = 6. ^*∗*^*p* < 0.05, ^*∗∗*^*p* < 0.01, compared with the normal group.

## Data Availability

The data used to support the findings of this study are included within the article.
